# Institutional Notes and News

**Published:** 1909-10-23

**Authors:** 


### Institutional Notes and News.

### HOSPITAL SATURDAY.

Special collections were made on Saturday last on behalf of the Hospital Saturday Fund, founded in 1873 for the purpose of obtaining help for the Metropolitan hospitals and kindred institutions from those who were not reached through the operations of the Metropolitan Hospital Sunday Fund. The fund has two main sources of income, one being a penny-a-week collection in factories and workshops—from which it derives about 70 per cent. of its present income of £30,000 a year—and the other annual collections on Hospital Saturday. Between 500 and 600 voluntary workers assisted on Saturday at railway stations, football grounds, and theatres and music-halls. Many of the metropolitan mayors rendered assistance by going to music-halls and other places of entertainment, and, where the management permitted it, making an appeal on behalf of the fund. Among those who assisted in this way were the mayors of St. Pancras, Hornsey, Lambeth, Greenwich, and Poplar. Collecting-boxes were also placed in many banks and business houses, though no collection was made in the streets, that mode of obtaining help having been discontinued since 1897. The committee of the fund has received several donations in response to the Lord Mayor's appeal, including one of £40 from the Consolidated Goldfields of South Africa, Ltd., and it is hoped that others will follow.

### WORCESTER GENERAL INFIRMARY.

At the monthly meeting of the Executive Committee, held on October 18, the resignation of Mr. T. Bates, who for thirty years has been Hon. Surgeon to the Infirmary, was received. Members of the Committee and honorary staff spoke expressing regret for his resignation and a high appreciation of his devoted services, and he was requested to consent to continue his association with the Infirmary in the capacity of Hon. Consulting Surgeon. A report was received from the House Committee on the subject of dealing with children suffering from gastro-enteritis. It was recognised that such children are suitable cases for hospital treatment and must be admitted, but that it is undesirable that they should be treated in the general children's ward. The report emphasised the need for separate wards for medical and surgical children, and of suitable provision for treating those suffering from phthisis in the open air. In the course of the discussion arising on this report, suggestions were thrown out as to the advisability of building a children's block in the ground adjoining the Infirmary, for which it would be necessary to make an appeal to the public. The matter was referred to the House Committee for further consideration. The report on the laundry and disinfection of linen was also submitted to the Committee, and it was realised that to carry out the suggested reforms will require considerable reconstruction of and additions to the present building. In view of the fact that the Secretary is about to visit Coventry Hospital and its laundry, which is said to possess the most modern equipment, this matter was postponed, awaiting his report.

### WORCESTER OPHTHALMIC HOSPITAL.

At the quarterly meeting of the Committee of this hospital the subject of the increased work thrown on the institution by the medical inspection of children attending the elementary schools was discussed. This hospital receives patients from the surrounding country districts as well as from the city. A recommendation was received from the hon. staff, advising the addition of an assistant surgeon to the staff to assist in this work. Acting upon this suggestion, the Committee appointed Dr. Neville Crowe, of Worcester. It was resolved by the Committee to suggest to the Elementary Education Authorities that they should subscribe through one of their members to the funds of the hospital, in return for which the department would receive letters of recommendation upon which the school children would be treated, placing them on the same footing as other patients.

### WEST SUFFOLK HOSPITAL.

Mr. and Mrs. Washington Charters, of the Manor, Horringer, have given new balconies at the West Suffolk Hospital, Bury St. Edmund's, which form a very desirable addition to the institution, enabling the medical staff to deal with cases which require open-air treatment in a manner which it has been impossible for them to do in the past. Although the balconies occupy the main front of the building, they do not act in any way to the disadvantage of the existing wards, either in blocking the light or in any other respect. The work of erecting the balconies was open to competition, and the design of Messrs. Boulton and Paul, Ltd., of Norwich, was adopted. The erection of the brick and stone sub-structure of the balconies was entrusted to Messrs. Hinnels and Son, of Bury St. Edmund's. In every detail the work has received the careful attention of the Hospital Committee, and their wishes have been ably interpreted by the contractors. The magnificent views to be obtained from the balconies over Hardwick and the neighbourhood, and the fine, bracing air, combine to make these additions to the hospital a lasting monument to their donors, and a boon, not only to the patients, but also the staff of the institution.

### NEW COTTAGE HOSPITAL, HOLYWELL.

Holywell's new cottage hospital was formally opened a fortnight ago. It is the gift to the town of Mr. Edwin Jones, J.P., of Clapham Park, London, who was born at Holywell in 1837, and who is the head of several large drapery concerns in London. With the new cottage hospital will be incorporated the Flintshire Dispensary, an institution started some 80 years ago. The hospital is situated on an elevated site at Penymaes, commanding lovely views, and is 400 feet above the sea level. The hospital has been properly furnished and equipped, and to this Mr. Randall Higgins (the donor's partner) has contributed the whole of the beds and bedding. The following gentlemen have been appointed trustees of the institution: The donor, the vicar of Holywell (for the time being), Messrs. J. Lloyd-Price, J.P., John K. Evans, J.P., Trevor Eyton, J.P., and H. A. Cope.

### BRADFORD EAR AND EYE HOSPITAL.

The rebuilding scheme of the Royal Ear and Eye Hospital, Bradford, an institution founded in 1857, provides for a new building several stories high. On the ground floor will be larger consultation and waiting rooms for out-patients, a small room for minor operations, together with sitting-room and bedroom accommodation for a resident house surgeon. Upon the first floor it is proposed to have a rearrangement of the present wards so as to secure two large new wards, male and female day rooms, and a pathological room. The second floor is to be devoted to several new wards, including a much-needed children's ward and an additional operating theatre, and the third floor to additional bedrooms for the nursing and domestic staff, bringing the sleeping accommodation for the resident staff from 17 beds to 35, while the number of patients' beds in the wards would be increased from 46 to upwards of 100. The architects, Messrs. Mawson and Hudson, have had considerable difficulty in enlarging the hospital on the present site to meet the urgent demands for such a large increase in the accommodation. It has been found necessary entirely to remodel the rear portion of the building, and to raise the front façade one story. The estimated cost of the alterations and extensions is £10,000, towards which an appeal is shortly to be made in Bradford and the larger area beyond, for the hospital is largely a county institution, serving practically the whole of the West Riding and even further afield. Whilst the eye department of the Leeds General Infirmary serves Leeds and the east of Yorkshire, there is no special hospital for eye and ear treatment between Bradford and Manchester, whilst in the north-westerly direction patients are often drawn from places as far distant as Lancaster and Morecambe.

### DERBYSHIRE CONVALESCENT HOME.

The Duke of Devonshire opened a bazaar at the Drill Hall, Derby, last week in aid of the Derby and Derbyshire Convalescent Home. The home is situated at Matlock Bank, and the president is Sir Henry Bemrose, who, in introducing the duke, mentioned that the institution was opened 20 years ago by his mother, Lady Edward Cavendish. Since then 13,000 patients have been admitted and something like £1,000 is at present needed to pay for some important additions and improvements which have recently been made. The Duke of Devonshire paid a high tribute to the work which the home carried on, and congratulated the management on their determination to increase its usefulness.

### NORFOLK AND NORWICH HOSPITAL.

The quarterly meeting took place last week in the Board Room of the institution. It was unusually well attended. Sir Peter Eade was called to the chair. Bishop Fisher was elected to serve on the Board of Management until April 1911, in the place of Lieut.-Colonel the Hon. H. W. Mansfield resigned. In response to an application from the Director-General of the Medical Department of the Navy the Board guaranteed the services of five fully trained and certificated nurses for duty in a naval hospital, if required, in the event of war. The question of providing a special department for dental cases was discussed at the meeting, and it was evident from the figures given that it is a very large question indeed, altogether too large for the hospital authorities to undertake at the present moment. A final decision was postponed till the next meeting.

